# On the Tests for Arsenic

**Published:** 1818-12

**Authors:** John Prideaux


*For the London Medical and Physical Journal.*

### *On the Tests for Arsenic.* By John Prideaux, Esq.

IT is unnecessary for me to say, that the case detailed by Mr. Hume in your last Number was determined in a manner perfectly convincing. It was to a confidence in the appearance of the precipitate, without subsequent analysis, that I principally objected. His mode of examination appears to be better than the metallic revival; being calculated to detect more minute quantities of arsenic. The expressions—"a mere atom," and "the very fraction of a grain," are indeterminate; but I have compared the two methods, and find that it would be difficult to obtain a demonstrative quantity of the metal from a precipitate, unless the solution from which it was produced contained as much as the thirtieth part of a grain of arsenic; whilst, by the sublimation and solution of the oxide, the precipitate from a solution containing a hundredth of a grain of that mineral may be proved to be arsenical. It occupies more time; but that, in matters of such importance, is not to be considered: yet, since it has generally happened, where such investigations have been instituted, that the quantity of arsenic present has considerably exceeded those above named, it is not to be wondered at if a mode which is easy of execution, and produces absolute certainty, has been continued without improvement. The most important object of my addressing you has, therefore, been answered, and I have gained an advantage by it, for which I am obliged to Mr. Hume; and I request that you, Gentlemen, will accept my thanks for the insertion of my letters.

On another point, however, we seem to be on worse ground than before. We must conclude, from Mr. Hume's reasoning on the case before us, that he has never succeeded to discover arsenic in the liquid contents of the stomach, on dissection. In this instance, he thinks the fluid contained none, though solid arsenic was present; and it would seem that, if it were swallowed in solution, it must have passed off long before death. We should thus be led to conclude, that it never will be found in that fluid; and hence an inference would follow, that, if a yellow precipitate appears in our examination of it, it is owing to some other substance. This is a most important consideration; and I hope no opportunities will be lost of putting it to the test of experiment.

In the case adduced, it is stated that the test gave no indication of arsenic in that liquid, and thence it is inferred there was none. But this inference is drawn from only one half of the premises. If we state the case thus—the liquid had stood twelve hours in contact with arsenic, while in Mr. Hume's possession, and, in all probability, more than twenty-four hours previously in the stomach of the deceased,—part of the time in an increased temperature; yet, on trial, the test indicated no arsenic;—I think the inference would be not quite so favourable to his conclusion, "that nothing is more easily detected than arsenic." It is in this fluid, and in compound or highly-coloured alimentary liquids, such as soups, porter, &c. that we may expect difficulty in the search. Of this any one may satisfy himself. A grain of arsenic, dissolved in three pints of distilled water, readily affects the test: let any one impregnate the fluid from a stomach with the mineral, in that proportion, and then try whether he can discover it by the silver-test, or any other means.

Since Mr. Hume has shown that he makes the analysis of the precipitate a part of his process, the methods of previously separating the phosphoric acid from the solution do not require so much attention. Otherwise, I should be disposed to observe, that the muriate of lime is more objectionable than the nitrate, as leaving an acid in the solution, which adds to the complexity and uncertainty of the process; and that nitrate of lead, when used in excess, which it is not easy to avoid, leaves an oxide, liable to the same disadvantage. Nitrate of barytes, in my opinion, answers better than either. But, though the phosphoric is among the easiest acids to separate from its soluble compounds, yet a method of getting rid of it, without injuring so delicate an operation as the testing for arsenic, is, I think, still a desideratum.

The ammoniaco-nitrate is certainly a very commodious form of applying the silver-test, and may save trouble in cases of little difficulty. In very compound cases, however,

I must still think its use will be rather injurious than useful. Phosphate of soda and arsenic will not often occur together, or be mistaken one for the other, except in the fluid from the stomach, where it is manifestly inapplicable. Ammonia seems to be preferable to subcarbonate of potass, as the alkali employed.

I am called on to prove my assertion, that Mr. Hume's query, with regard to onions, is at least as applicable to arsenic; and I shall give my reasons in the briefest manner I am able, as they do not materially affect the subject of my letter. If there was any satisfactory demonstration of the peculiar attraction between white arsenic and the villi of the stomach, which is proof against vomiting, &c. as supposed by Mr. Hume, I should certainly agree that onions would be more likely to pass off. But I have not observed such an attraction. In the stomachs of dogs, to which arsenic had been given, in substance, in one case to the extent of ten grains, and in another of half a drachm, and had produced vomiting, none of the mineral could be found; though the animals were killed, and their stomachs examined, within twenty-four hours after the poison had been swallowed: and the corrugated form of the inner coat of that organ in the dogs would seem particularly favourable to its detention: but undigested food was found in small quantity. In Mrs. Downing's case, onions had been swallowed, containing, of course, a portion of the liquid in which they were boiled. I apprehend this will bear out my opinion.

If Mr. Hume will repeat my experiment, where he attributes the error to excess of alkali, he will perhaps find the same results as I did, without either excess or error. I conceive the deep blue, with copper, to be characteristic of ammoniac: I thought the muriate the most likely salt to be present; and I found a weak solution of muriate of ammonia produce a similar effect. Hence, I inferred the presence of that salt. I certainly said nothing about a precipitate, because there was none.

The paragraph on the subject of diplomas, &c. I suppose to be ironical; but, if it be serious, I must give Mr. Hume the trouble to refer once more to my letter, where he will see that it does not apply to my objection. It is one thing to estimate the competency of a witness, and another to understand the reasoning on which he founds his opinion; and it is the difficulty of the latter which makes the former necessary. This brings us again to the trial at Launceston. I feel, with Mr. Hume, the difficulty of handling the subject with propriety; but there are three points which I wish to be observed;—1. The jury were not chemists. 2. The experiments *detailed* to demonstrate the existence of arsenic, were very simple. 3. The quantities of precipitate were exceedingly minute.—These features, considered with a little attention, will, I think, sufficiently express my reasons for regarding the experiments of the defence as properly applicable to the purpose.

There is a very important remark in Mr. Hume's last letter, on which I have yet to touch. A minute portion of a drop of ammoniaco-nitrate of silver, applied to the inside of a coffee-cup, from which the deceased had drunk, gave, when examined by the magnifier, indications of arsenic; and *this proof alone* would be sufficient to warrant my proceeding to an inquest."—Is this any proof at all? Suppose she had drunk from this cup a dose of phosphate of soda, acidulated with lemon or vitriol,—not an uncommon mode of taking salts, to obviate its nauseous flavour, &c.—the application of the ammoniaco-nitrate, unless in a concentrated state, would quickly have produced a yellow spot. It is true the phosphate becomes brown sooner than the arsenite, under such circumstances: but is this a ground for proceeding to an inquest? However dense the yellow, and flocculent the precipitate, it is not, in my opinion, sufficient proof of its being arsenical to justify any evidence which could influence the decision of a coroner's jury, or of any other; and I am not a little surprised at the expression I have quoted, after the assertion in a former letter of Mr. Hume's, that he would not swear to arsenic from the results of one experiment; and that, whenever he failed to satisfy his own mind, his evidence should lean to the favourable side.

*Plymouth; Oct.* 14, 1818.

